# SOLD1 is expressed in bovine trophoblast cell lines and regulates cell invasiveness

**DOI:** 10.1186/1477-7827-12-55

**Published:** 2014-06-21

**Authors:** Mahmoud Awad, Katsuo Koshi, Keiichiro Kizaki, Toru Takahashi, Kazuyoshi Hashizume

**Affiliations:** 1 Laboratory of Veterinary Physiology, Cooperative Department of Veterinary Medicine, Faculty of Agriculture, Iwate University, 3-18-8 Ueda, Morioka, Iwate 020-8550, Japan; 2 United Graduate School of Veterinary Science, Gifu University, Gifu, Japan; 3 Tokyo Metropolitan Institute of Gerontology, Tokyo, Japan; 4 Cooperative Department of Veterinary Medicine, Faculty of Agriculture, Iwate University, Morioka 020-8550, Japan

**Keywords:** SOLD1, Placenta, Trophoblast, Ly-6 domain, Cell invasion

## Abstract

**Background:**

Secreted protein of Ly-6 domain 1 (SOLD1), a secretory-type member of the Ly-6 superfamily, is expressed in both fetal and maternal tissues throughout gestation. SOLD1 mRNA is expressed in the endometrium and in trophoblast mononucleate and binucleate cells, suggesting it plays an important role not only in placental architecture at early gestation, but also in remodeling the endometrium at late gestation. Here, we investigate the expression of SOLD1 mRNA and protein in trophoblast cell lines. In addition, we examine the effect of SOLD1 on the invasive ability of trophoblast cells.

**Methods:**

We measured *SOLD1* gene expression in thirteen bovine trophoblast (BT) cell lines by using quantitative reverse transcription PCR (qRT-PCR). SOLD1 protein levels were examined in two cell lines, BT-C and BT-K, by using Western blotting and immunocytochemistry. In addition, we measured the invasive activity of BT cells in the presence or absence of anti-bovine SOLD1 antibodies.

**Results:**

At variable levels, SOLD1 was expressed in all thirteen cell lines; however, expression remained below that of proximal fetal membrane tissue. SOLD1 protein, which was approximately 28 kDa in size, was detected in perinuclear area of the cytoplasm in BT cells. Treatment with anti-bovine SOLD1 antibody had a dose-dependent suppressive effect on the invasiveness of BT-K cell lines.

**Conclusions:**

The present study is the first to investigate SOLD1 expression in vitro, in trophoblastic cell lines. Our data suggested that SOLD1 is involved in the regulation of the trophoblast invasiveness. Therefore, SOLD1 may play an active and crucial role in mediating communication at the fetomaternal interface.

## Background

Trophoblastic cells, which arise from the outer layer of blastocyst, are thought to be the first cells to differentiate during embryogenesis [1]. Trophoblast is considered one of the basic fetal compartments that contribute to the formation of the placenta. Bovine placenta is synepitheliochorial, directly contacting maternal endometrial cells at limited sites called placentomes [2]. Bovine trophoblast cells are categorized into two main types, trophoblast mononucleate cells (TMCs) and trophoblast binucleate cells (BNCs) [3,4]. BNCs arise from the differentiation of TMCs through a cytokinetic mitotic division [4,5]. Both cell types play a key role during the peri-implantation period by secreting various cell type-specific molecules. For example, interferon-τ (IFN-τ) [3] is produced by TMCs, and BNCs secrete BCL2-related protein A1 (BCL2A1), an anti-apoptotic factor involved in cell maintenance [6], placental lactogen (CSH1) [7], prolactin-related proteins (PRPs) [8], and pregnancy-associated glycoproteins (PAGs) [9]. Together, these molecules are important for embryo implantation, maintaining pregnancy, and fetal growth.

Bovine SOLD1, a secreted member of Ly-6 superfamily, has a Ly-6 domain (lymphocyte antigen-6, Ly-6/urokinase-type plasminogen activator receptor (uPAR)) that consists of 70–100 amino acids characterized by a conserved pattern of 8–10 cysteine residues and a defined pattern of disulfide bonding [10-13]. Previous studies showed that SOLD1 is detected in TMCs at early and mid-gestation, and detected in both TMCs and BNCs at late gestation. SOLD1 protein is distributed throughout the villous tree mesenchyme in placentomes and its expression increases in the endometrial epithelium and sub-epithelial stroma as pregnancy progresses, suggesting that SOLD1 plays a key role in placental construction, fetal growth, and fetal membrane expansion during early pregnancy [10,12,13]. In addition, SOLD1 is likely involved in the remodeling of the endometrium during late pregnancy [10]. However, the actual physiological function of SOLD1 and the mechanism by which SOLD1 is regulated, remain unclear.

Trophoblast cell lines are considered a convenient tool to elucidate the function of trophoblast-specific molecules that are predicted to play a crucial role during pregnancy. Trophoblast cell lines can also be used to clarify the mechanisms that regulated the function of trophoblast-specific factors. Furthermore, the cell lines can be used to learn more about the cellular characteristics of trophoblast. For these purposes, several cell lines have been established from different species including pigs, mice, and cows [14-18].

For the implantation process to be successful in ruminants, three different steps must be achieved. First, the conceptus elongates prior to implantation. Next, the fetal trophoblastic cell layer opposes and adheres closely to the maternal endometrial epithelial layer. Lastly, the trophoblast cells invade into the endometrial epithelium, resulting in the fusion of maternal and fetal tissues and the initiation of placenta formation [19-21].

The invasion of fetal trophoblast into maternal uterine tissues in humans and mice is considered a critical process in fetal development, providing adequate fetomaternal fixation [22,23]. Throughout bovine gestation, BNCs represent approximately 20% of the fetal chorionic epithelium. BNCs migrate to the opposed uterine epithelium without passing through the basement membrane. At the interface, BNCs release their granules into uterine epithelium [24,25]. In contrast, in sheep, there is restricted invasion that results in the formation of syncytia [26]. In the present study, we investigated the levels of SOLD1 mRNA and protein in BT cell lines, and examined the effect of SOLD1 on the invasiveness of trophoblastic cells in vitro.

## Methods

### Cell culture

Thirteen BT cell lines (BT-1 and BT-A–L) were established from in vitro matured and in vitro fertilized blastocysts as described previously [18,27]. The BT cell lines were cultured and maintained according to a previously described method [28]. In brief, the cells were cultured in Dulbecco’s modified Eagle’s medium/F-12 medium (Sigma, Saint Louis, MI, USA) containing 100 IU/ml of penicillin and 100 μg/ml of streptomycin (Sigma), supplemented with 10% fetal bovine serum (FBS; Biowest, Nuaillé, France), at 37°C in an atmosphere of 5% CO_2_. The medium was changed every 2–3 d. A monolayer of confluent BT cells was mechanically passaged by pipetting. Collagen-coated flasks were prepared by incubating flasks with acid-soluble porcine type I collagen (3 mg/ml of type I-C collagen; Nitta Gelatin Osaka, Japan), diluted 10-fold with distilled water and poured into flasks for more than an hour. Flasks were then washed with general culture medium. After a phosphate-buffered saline (PBS) wash, dissociated cell aggregates were plated in the collagen-coated flasks. The cell cultures grown in collagen-coated flasks were used for RNA purification, Western blotting, or immunocytochemistry. BT-1 was used as a standard cell line because it was maintained and monitored for more than 300 passages, and may show features of stable trophoblastic cells.

### RNA extraction and RT-PCR

Total RNA was isolated from the cultured cells and bovine proximal fetal membranes (PFM) using TRIzol Reagent (Invitrogen, Carlsbad, CA, USA) according to the manufacturer’s instructions. The yield of total RNA was quantified by measuring the absorbance at 260 nm (A_260_). RNA quality was determined by measuring the A_260_/A_280_ ratio using a NanoDrop spectrophotometer (ND-1000, Wilmington, DE, USA) and by using 1% agarose gel electrophoresis. Genomic DNA was removed using DNase and the TURBO DNA-free Kit (Ambion, Austin, TX, USA). Two micrograms of total RNA was reverse-transcribed into cDNA using random primers and a high-capacity reverse transcription kit (Applied Biosystems, Foster City, CA, USA) according to the manufacturer’s instructions. The reverse transcription (RT) cycle conditions were as follows: 25°C for 10 min, 37°C for 120 min, and 85°C for 5 s. The resultant cDNA was stored at −20°C. Each PCR reaction contained cDNA template, primers (Table 1), and AmpliTaq Gold PCR Master Mix (Applied Biosystems). The PCR reaction was performed as follows: 95°C for 30 s for denaturation, 57°C for 30 s for annealing, and 72°C for 1 min for extension, repeated for 26 cycles. The PCR products were analyzed using agarose gel electrophoresis and visualized by ethidium bromide staining. The amplified products were subcloned into the pGEM-T Easy vector (Promega, Madison, WI, USA) and sequenced using a BigDye Terminator cycle sequencing kit and an automated sequencer (Applied Biosystems).

**Table 1:** Primers used for RT-PCR analysis

**Gene**	**Primer**	**Sequence**	**Position**
***SOLD1*** [NM_001105478]	F	5′-TCCAGAGATGGCTAAGTGCCTT-3′	50–71
	R	5′-GAGTTGGACATGACTGAGCCAC-3′	453–432
***GAPDH*** [NM_001034034]	F	5′-CCTTCATTGACCTTCACTACATGGTCTA-3′	173–201
	R	5′-GCTGTAGCCAAATTCATTGTCGTACCA-3′	1029–1002

### Quantitative RT-PCR

The level of SOLD1 mRNA in the BT cell lines and PFM were confirmed by qRT-PCR by using a SYBR Green assay (Applied Biosystems) as described previously [29]. The primer pairs used for qRT-PCR are described in a previous study [29] (Table 2). The relative differences in the initial levels of each cDNA species were determined by comparing their threshold cycle (Ct) values. To quantify the mRNA copy number, a standard curve was generated for each gene by using serial dilutions of plasmids containing the corresponding cDNAs. The dissociation curve used to detect the SYBR green-based amplicons of interest was accurate, because SYBR green also detects double-stranded DNA, including primer dimers, contaminating DNA, and PCR products from misannealed primers. Contaminating DNA and primer dimers appeared as separate peaks from the desired amplicon peak. For each gene, the ratio of its mRNA expression level to that of glyceraldehyde-3-phosphate dehydrogenase (*GAPDH*) was calculated to adjust for variations among the qPCR reactions. All values are presented as the mean ± standard error of the mean (SEM).

**Table 2:** Primers used for qRT-PCR analysis

**Gene**	**Primer**	**Sequence**	**Position**
***SOLD1*** [NM_001105478]	F	5′-GGAAGCACCTGCCAGACTCA-3′	177–196
	R	5′-AAAGCGTGCCATTTTCGAAG-3′	246–227
***TNF-α*** [NM_174662]	F	5′-AAGCCGGTAGCCCACGTT-3′	297-314
	R	5′-TGAGGGCATTGGCATACGA-3′	375-357
***BCL2A1*** [NM_001037100]	F	5′-TTGCAGATACAGCAACCTGGAT-3′	73-94
	R	5′-GGACAGAGGAAGCCACATCTTG-3′	145-124
***GAPDH*** [NM_001034034]	F	5′-AAGGCCATCACCATCTTCCA-3′	280–299
	R	5′-CCACCACATACTCAGCACCAGCAT-3′	355–332

SOLD1, secreted protein of Ly-6 domain 1; TNF-α, Tumor necrosis factor α; BCL1A1, Bcl-2-related protein A1; GAPDH, glyceraldehyde 3-phosphate dehydrogenase; F, forward; R, reverse; qRT-PCR, quantitative reverse transcription polymerase chain reaction.

### Western blot analysis

Western blots were performed as previously described [18]. SOLD1 protein was extracted from BT-C and BT-K cell lines (cell lysates and/or conditioned media). Briefly, cells were cultured until they reached confluence and washed using serum-free media, followed by PBS. Cells were homogenized in lysis buffer containing 50 mM Tris (pH 7.5), 150 mM NaCl, 0.1% Triton X-100, and Complete, Mini protease inhibitor cocktail (Roche Diagnostic GmbH, Mannheim, Germany). After centrifugation at 15800 × *g* for 15 min at 4°C, the supernatant was collected and reserved for further analysis. BT-C and BT-K cell lines were cultured on cell culture inserts (8-μm pore size, BD Biosciences) in 12-well plates. Cells were incubated in serum-free medium in the upper and lower chamber for 48 hrs. The upper and lower conditioned media were collected and cold acetone was added (1:4). After an overnight incubation at −30°C, the conditioned media was centrifuged and the supernatant was immediately replaced with PBS to dissolve the protein. The concentration of total protein from cell lysates and conditioned media was analyzed using the Quick Start Bradford Protein Assay Kit (Bio-Rad Laboratories, Hercules, CA, USA). The proteins of the cell lysate and conditioned media (8 μg and 3 μg, respectively), were separated using 12% sodium dodecyl sulfate polyacrylamide gel electrophoresis and transferred to PVDF membranes (Immobilon-P, Millipore Corporation, Bedford, MA, USA). The membranes were blocked with 10% skim milk overnight at 4°C and incubated with custom-made bovine anti-bSOLD1 antibody (1:1000) [12] for 1 h at room temperature. Membranes were subsequently incubated with alkaline phosphatase-conjugated anti-rabbit IgG (1:3000; Sigma) for 1 h at room temperature. An alkaline phosphatase detection system (Bio-Rad Laboratories) was used to detect immunoreactive SOLD1.

### Immunocytochemistry

BT-C and BT-K cells were cultured for 8 d in covered 2-well Lab-Tek Chamber slides (Lab-Tek, #177429) precoated with collagen (as above). After the cells had reached confluence, the slides were fixed using 4% paraformaldehyde in 0.1 M PBS (pH 7.4) for 30 min and then incubated with anti-SOLD1antibody (1:200) [12] or normal rabbit serum for 2 hrs at room temperature. After being washed in PBS with Triton X-100 (PBST), the slides were incubated with secondary antibody (Alexa Fluor 488 donkey anti-rabbit IgG; 1:1000 in PBST; Invitrogen). To visualize the nuclei, the slides were mounted in Dako fluorescent mounting medium (Dako North America, Inc., Carpinteria, CA) after stained with Hoechst 33342 (Invitrogen). The immunoreactive signals were examined using an ECLIPSE 80i microscope (Nikon, Tokyo, Japan).

### Invasion assay

BT-K and BT-C cell invasion was assessed using a 24-well plate BD BioCoat Matrigel Invasion Chamber (BD Biosciences, Bedford, MA). Inserts contained polyethylene terephthalate membranes (8-μm pore size) coated with a thin layer of Matrigel and a reconstituted basement membrane that prevented non-invasive cells from migrating through the pores of the membrane. Invasive cells were able to detach, invade, and migrate through the Matrigel-coated membrane. Experiments were performed according to manufacturer’s instructions. BT-C (2.42 × 10^4^) and BT-K cells (2.25 × 10^4^) were trypsinized, counted, and resuspended in serum-free media. To each well, 750 μl of medium containing 10% FBS (the chemoattractant) was added. In the upper well, 500 μl of cell suspension was loaded. The plate was incubated for 6 d at 37°C in humidified air containing 5% CO_2_. After the incubation period, the non-invading cells were removed from the upper surface of the filters by using a cotton-tip applicator. The cells on the lower surface of the Matrigel were fixed in 100% methanol, stained using 0.5% hematoxylin, and counted in 4 random high-power (40×) fields using an ECLIPSE 80i microscope (Nikon, Tokyo, Japan). This procedure was repeated in the presence of anti-bSOLD1 antibody. Anti-bSOLD1 antibody (1:100, 1:500, and 1:1000) was added to the serum-free media in the upper part of the Matrigel invasion chamber.

### Statistical analysis

The values are presented as the mean ± SEM. The qRT-PCR was performed twice for each animal sample, and each experiment was performed in triplicate. All qRT-PCR data were analyzed using one-way analysis of variance, followed by the Turkey-Kramer test or *t*-test. JMP 7 software (SAS Institute Inc, Cary, NC, USA) was used for the analysis. Differences were considered significant at P < 0.05.

## Results

### SOLD1 mRNA expression in bovine trophoblast cell lines

**Figure 1:**
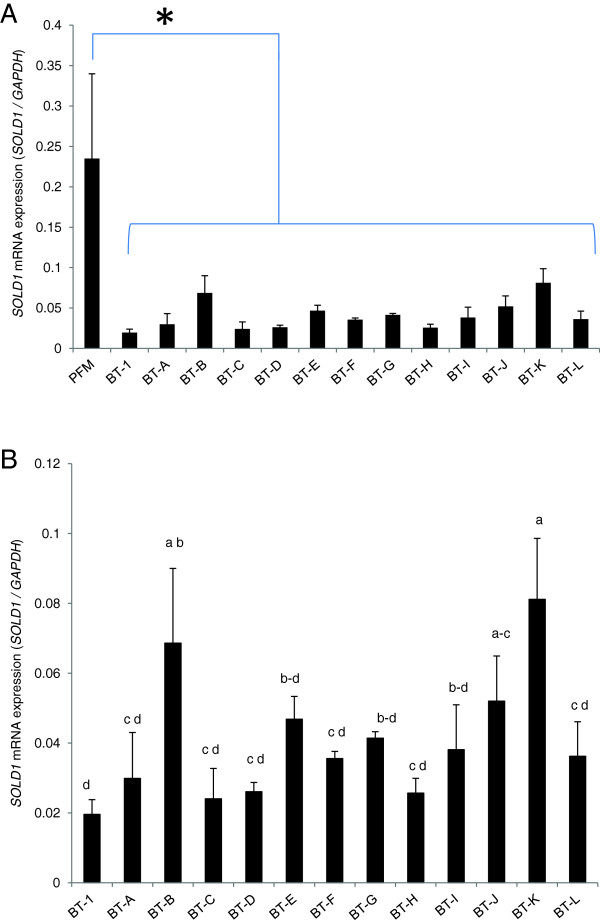
**Expression of *SOLD1 *mRNA. (A)** The expression levels of secreted protein of Ly-6 domain 1 (SOLD1) in bovine trophoblast (BT) cell lines (BT-A–L) in comparison with those in proximal fetal membranes (PFM), an in vivo control, and in BT-1, which was previously established, were analyzed by quantitative reverse transcription PCR (qRT-PCR). **(B)** The expression level of SOLD1 in BT cell lines. Expression of these mRNAs was normalized to the expression of GAPDH measured in the corresponding RNA preparation. The bar graph shows the means ± SEM values. Bars labeled with an asterisk **(A)** and different letters **(B)** are significantly different from each other (P < 0.05).

The expression of SOLD1 mRNA was analyzed in thirteen BT cell lines and PFM samples by using qRT-PCR. Every BT cell line expressed SOLD1 mRNA, but at variable levels. Notably, the expression level of SOLD1 in all the BT cell lines tested was below that in PFM samples (Figure 1A). With the exception of BT-1, the BT-K and BT-C cell lines showed the highest and lowest expression of SOLD1, respectively (Figure 1B).

### Localization of SOLD1 protein in BT cell lines

**Figure 2:**
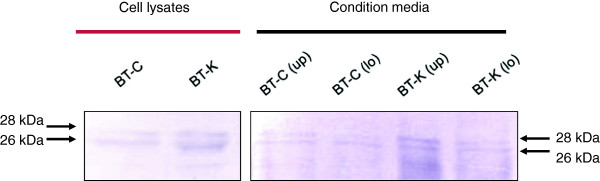
**SOLD1 protein levels in BT-C and BT-K cell lines.** The upper (up) and lower (lo) conditioned medium and BT-C and BT-K cell lysates were analyzed by Western blot. Arrows indicate SOLD1 bands that are 26 kDa and 28 kDa in size.

**Figure 3:**
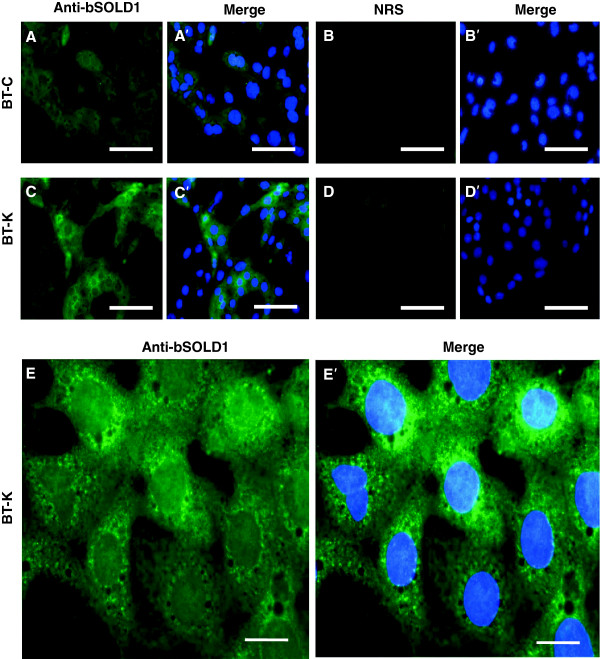
**Immunohistochemical localization of SOLD1 protein in BT cell lines.** Immunoreactive protein was detected in BT-C and BT-K using an anti-bovine SOLD1 antibody (anti-bSOLD1) **(A, A’, C, and C’)**. Normal rabbit serum (NRS) was used as a control **(B, B’, D, and D’)**. Nuclear DNA was stained with Hoechst 33342 **(A’–D’)**. Scale bars = 50 μm. Higher magnification images of BT-K cells **(E and E’)** were obtained using an oil immersion lens, scale bars = 10 μm.

SOLD1 protein was detected in both BT-C and BT-K cell lines by using Western blotting and immunocytochemistry. SOLD1 protein (approximately 28 kDa) was evident in the lysates and upper/lower conditioned media of both cell lines (Figure 2). Moreover, immunostaining showed that SOLD1 protein was localized to the perinuclear area of the cytoplasm in BT-C and BT-K cells (Figure 3).

### The effect of anti-bSOLD1 antibody on the invasiveness of BT cell lines

**Figure 4:**
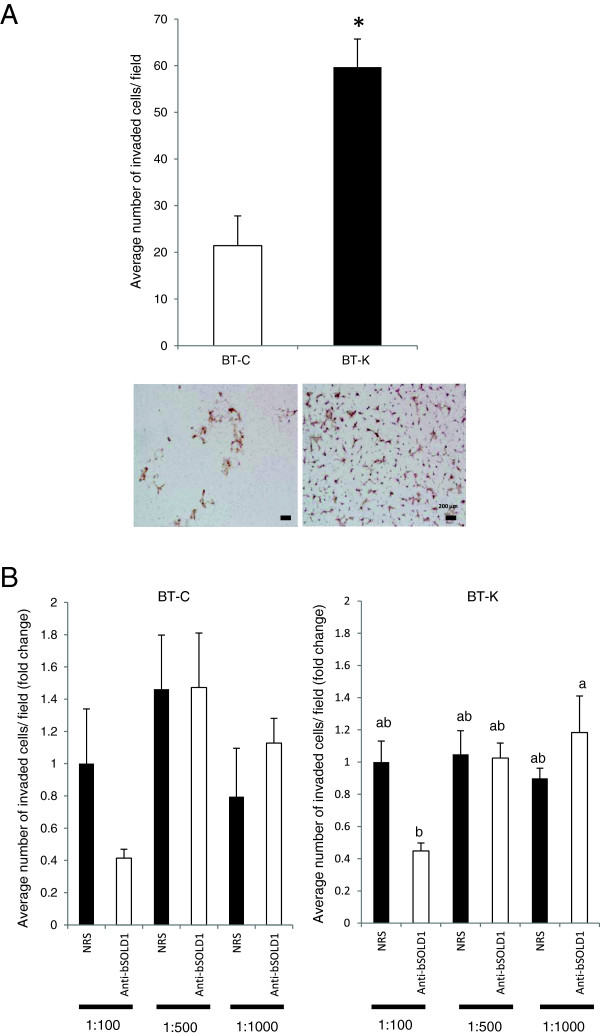
**Invasiveness of BT-C and BT-K cells. (A)** The invasive ability of BT-C and BT-K cells was measured using Matrigel invasion chamber assay. Hematoxylin staining was used for staining the invaded cells. Scale bars = 200 μm. **(B)** The invasion of BT-C and BT-K cells treated with different dilution of anti-bSOLD1 antibody (1:100, 1:500, and 1:1000). Fold change was calculated by dividing each value by the average value in NRS (1:100). The asterisk **(A)** and different letters **(B)** indicate values that are significantly different from each other (P < 0.05).

We examined the invasive ability of two BT cell lines, BT-C and BT-K. BT-K cells were found to have greater invasiveness than BT-C cells (Figure 4A). To investigate whether there was a relationship between the invasiveness of the BT cells and SOLD1 expression, BT-C and BT-K were treated with anti-bSOLD1 antibodies at three different dilutions. The data showed that no significant differences existed between the untreated groups and their corresponding treated groups for either cell line. Interestingly, anti-bSOLD1 antibodies significantly suppressed the invasive ability of BT-K but not BT-C cells at a high concentration (1:100) in comparison to the other treated groups. The suppression of cell invasiveness of BT-K cells by anti-bSOLD1 was dose-dependent (Figure 4B).

## Discussion

To our knowledge, the present study is the first to report the expression of SOLD1 mRNA and its protein localization in BT cell lines. Furthermore, this study is the first showing the effect of SOLD1 on the invasive ability of BT cell lines. Our results revealed that the BT cells showed different levels of SOLD1 mRNA. BT-K and BT-C cell lines, other than BT-1, that expressed the highest and lowest SOLD1 mRNA levels, respectively, were used for subsequent analyses. SOLD1 protein was detected in the perinuclear area of the cytoplasm in both BT-C and BT-K cell lines. Lastly, BT-K cells showed greater invasive ability than BT-C cells and the invasiveness of BT-K cells was suppressed by anti-bSOLD1 antibody treatment.

Trophoblast cells that line the external surface of the fetal chorioallantois are derived from the outer blastomeres and can be divided into two distinct populations, TMCs and BNCs [3,24,30]. Both cells secrete several important molecules that are important for implantation and placenta formation. TMCs produce IFN-τ [31-33], the maternal recognition molecule of pregnancy. BNCs, which differentiate from TMCs by karyokinesis without cytokinesis [34] or endoreduplication [28,35], represent approximately 15–20% of the total trophoblast population throughout gestation [36]. BNCs secrete various molecules including SOLD1, progesterone, estrogen, CSH1, PRPs, PAGs and others [10,37-41]. In cows, sheep, and goats, BNCs function primarily at the fetomaternal border, where they migrate from the fetal trophoblast to the uterine epithelium and fuse to form hybrid cells or syncytia [4,5,36]. This process is required to connect fetal compartments to the maternal circulatory system and to allow BNCs to release their cytoplasmic granules, which contains mediators, (e.g. placental lactogen) directly to the maternal side [4].

Analysis of the fetomaternal interface around the time of implantation is crucial to ensure the success of pregnancies; however, the mechanisms underlying fetomaternal interactions remain unclear. In vitro methods may serve as a tool for examining this complex interaction. Trophoblast cells are essential for fetal development and the maintenance of pregnancy in numerous species [14,16,17,27,42-46]. Recently, we developed BT cell lines derived from in vitro fertilized (IVF) blastocysts using bone morphogenetic protein 4. These cells were maintained for approximately 50 passages and exhibited characteristic trophoblast features. According to trophoblast gene expression profiles, these BT cell lines expressed the BNC-specific factors CSH1, PAG, and PRP, the TMC marker IFN-τ, and SOLD1 [10,12,13]. Various molecules derived from trophoblastic cells have been examined in a number of studies; however, information pertaining to SOLD1 remains limited [10,12,13]. SOLD1 belongs to the Ly-6 superfamily of secreted factors and is exclusively found in the female reproductive system and placenta [12,13]. We found that SOLD1 mRNA was present in all BT cell lines, but at different levels. These data are in accordance with previous studies showing that SOLD1 was detected in both trophoblast cell types [10]. In previous studies, SOLD1 protein was detected in BT-1 cell lysates, but not in conditioned media when cells were cultured on collagen-coated plates [12]. In contrast, we detected SOLD1 protein, in cell lysates and in conditioned media (upper and lower) from BT-C and BT-K cell lines by using an insert culture system. We attribute these conflicting results to the fact that SOLD1 has specific binding to the telopeptide in fibrillar type I collagen and, to a lesser extent, towards the reticular type III collagen [12]. SOLD1 secreted from trophoblast cells directly binds to the collagen coating of typical culture plates; however, we used an insert culture system that allowed SOLD1 protein to remain free in the conditioned media. SOLD1 immunoreactive signals were localized to the perinuclear area of the cytoplasm in both BT-C and BT-K cell lines, a pattern similar to that of other family members [47].

SOLD1 mRNA levels were not changed by culturing cells on different substrates (collagen coat, collagen gel and Matrigel) for a more than a week (8 d) (Additional file 1: Figure S1A). However, Matrigel stimulated expression in long-term cultures (20 d), but not in the presence of collagen coat and collagen gel (Additional file 1: Figure S1B). Matrigel contains various cytokines, and supplies a suitable environment for cellular activities [48]. Although these different phenomena (long-term culture on different matrices) implicate the involvement of apoptotic process, the expression of the apoptosis-related molecules, tumor necrosis factor α (TNF-α) and BCL2A1, showed no relation to SOLD1 expression even after 3 weeks of culture. In particular, Matrigel culture maintained cell activities and induced differentiation, as indicated by the expression of CSH1, PRPs, PAGs [49] (Additional file 2: Figure S2). Based on that, we hypothesized that SOLD1 is regulated by some cytokines and scaffold conditions; however, the effect of Matrigel on SOLD1 expression remains to be examined. Other specific features of trophoblastic cells are migration and invasion, which are crucial and characteristic placental processes in different species, including humans, mice [23], and horses [50]. During placentation in ruminants, including cows, trophoblastic cells first become apposed to the endometrial epithelia, and then BNCs migrate to the maternal side and fuse with epithelia [41].

In the present study, we found that SOLD1 induced trophoblast invasion. Its activity may be supported by the trophoblastic cells themselves and maternal side production of SOLD1, as suggested by our previous work showing that SOLD1 is detected in cells on both sides of fetomaternal interface [10]. Several growth factors, including fibroblast growth factor 7 and its receptor, have been detected in fetal trophoblast, suggesting paracrine cell-cell interactions [51]. We hypothesized that SOLD1 becomes localized on both sides of the interface through a similar mechanism. Alternatively, SOLD1 might cross to the maternal side through migration and restricted invasion of BNCs that fuse with the uterine epithelium, forming hybrid cells. In our study, the invasion assay revealed that SOLD1 is involved in the regulation of BT cell invasiveness.

## Conclusions

We have analyzed the expression level of SOLD1 in BT cell lines, a suitable in vitro model for bovine placental trophoblast. SOLD1 was expressed by all the BT cell lines we examined, but at variable levels. In the BT cell lines, the expression level SOLD1 was below that of fetal membrane tissues. Excluding the formerly established BT-1 cell line, BT-K and BT-C cells had the highest and lowest SOLD1 levels, respectively, of the lines examined. In a dose-dependent manner, anti-bSOLD1 antibodies suppressed the invasiveness of BT-K but not of BT-C cells. Together, these data suggested that SOLD1 is involved in the regulation of trophoblast migration and invasion at the fetomaternal interface.

## Competing interests

The authors declare that they have no competing interests.

## Authors’ contribution

MA planned the study, carried out experiments, and drafted the manuscript. KaK helped in performing the analysis. Ke.K. collected samples and conducted experiments. TT collected samples, raised the anti-SOLD1 antibody, and contributed to discussions. KH designed the experiments, prepared samples, and helped to write the manuscript. All authors read and approved the final manuscript.

## Supplementary Material

Additional file 1: Figure S1 *SOLD1* expression in cells cultured on different substrates. (A) BT-C and BT-K were cultured on three different substrates, collagen coat (control), collagen gel, and Matrigel, for the normal culture period (8 d). (B) BT-C cells were cultured on the same substrates for a longer time (20 d). The results were normalized to GAPDH mRNA expression and represented as fold-change values, calculated by dividing each value by the average value on day 4. The data shows the means ± SEM. The black bars, gray bars, and white bars represent the control, collagen gel, and Matrigel cultures, respectively. The different letters indicate a significant difference compared with the control (P < 0.05). Click here for file

Additional file 2: Figure S2 *TNF-α* and *BCL2A1* expression on different substrates. BT-C was culture on three different substrates, collagen coat (control), collagen gel, and Matrigel, for 20 days. Expression levels of tumor necrosis factor α (TNF-α) and BCL2-related protein A1 (BCL2A1) were normalized to the expression of GAPDH, measured in the corresponding RNA preparation. The black bars, gray bars, and white bars represent the control, collagen gel, and Matrigel cultures, respectively. The different letters indicate a significant difference compared with the control (P < 0.05). Click here for file
