# Supplementary figures and images for: SOLD1 is expressed in bovine trophoblast cell lines and regulates cell invasiveness
Source: Reprod Biol Endocrinol. 2014 Jun 21;12:55. doi: 10.1186/1477-7827-12-55 (PMC4078357; doi:10.1186/1477-7827-12-55)

**A**

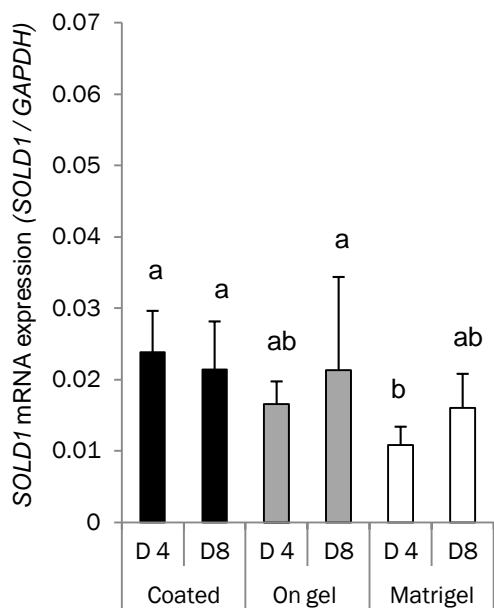

BT-C

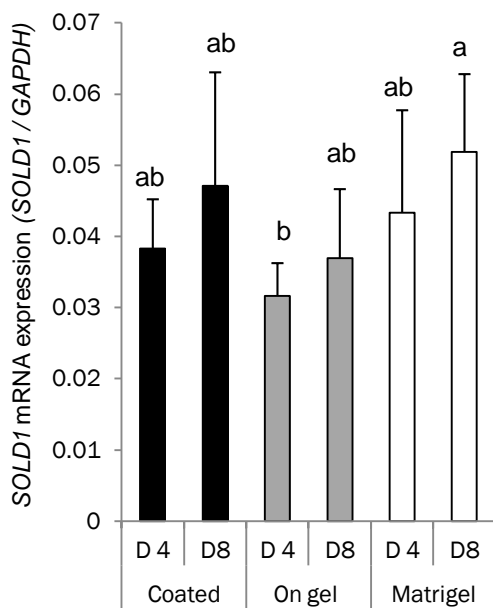

BT-K

**B**

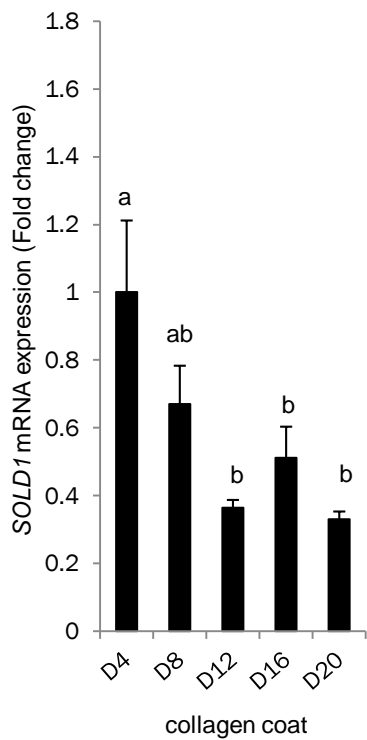

collagen coat

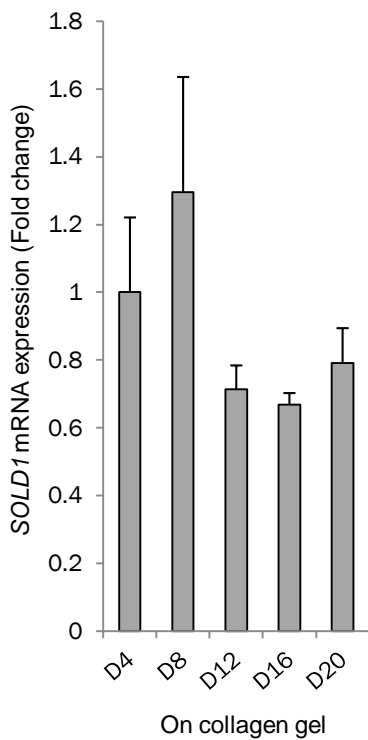

On collagen gel

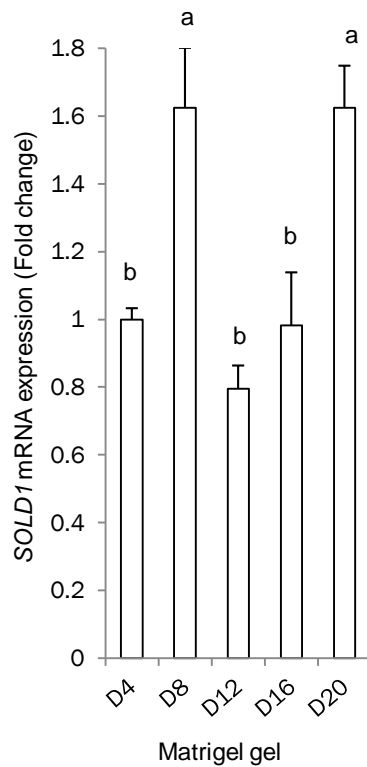

Matrigel gel

BT-C

Supplement: Additional file 1: Figure S1 — SOLD1 expression in cells cultured on different substrates. (A) BT-C and BT-K were cultured on three different substrates, collagen coat (control), collagen gel, and Matrigel, for the normal culture period (8 d). (B) BT-C cells were cultured on the same substrates for a longer time (20 d). The results were normalized to GAPDH mRNA expression and represented as fold-change values, calculated by dividing each value by the average value on day 4. The data shows the means ± SEM. The black bars, gray bars, and white bars represent the control, collagen gel, and Matrigel cultures, respectively. The different letters indicate a significant difference compared with the control (P < 0.05). [file 1477-7827-12-55-S1.pdf]

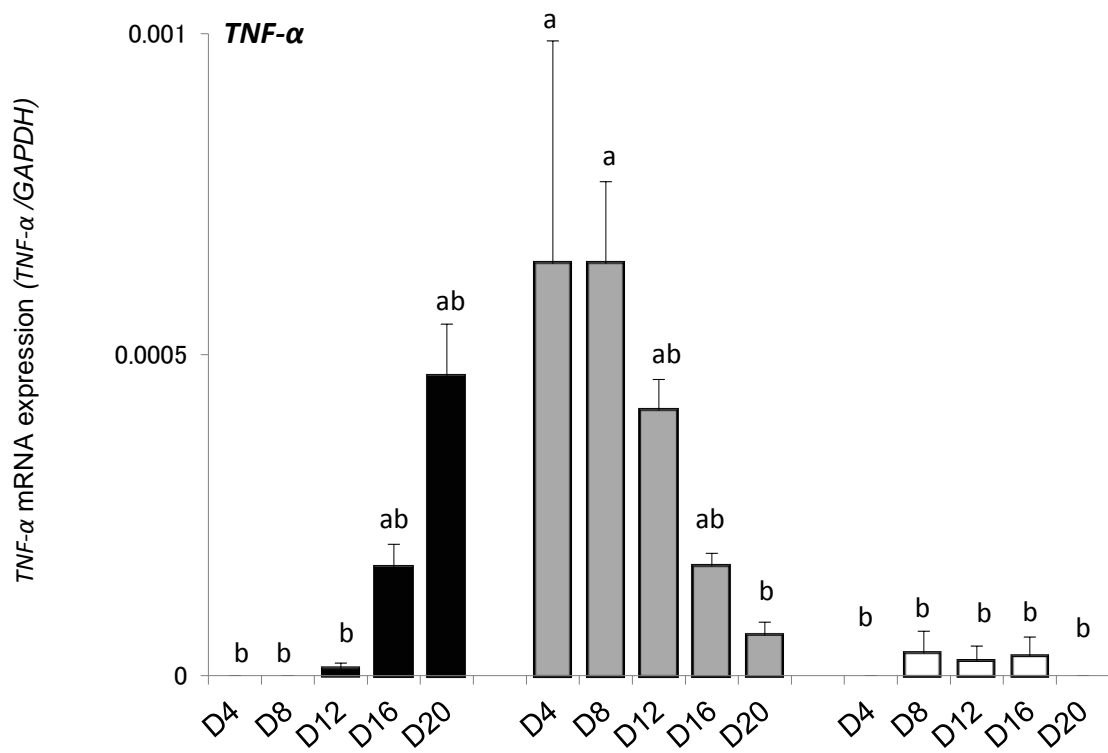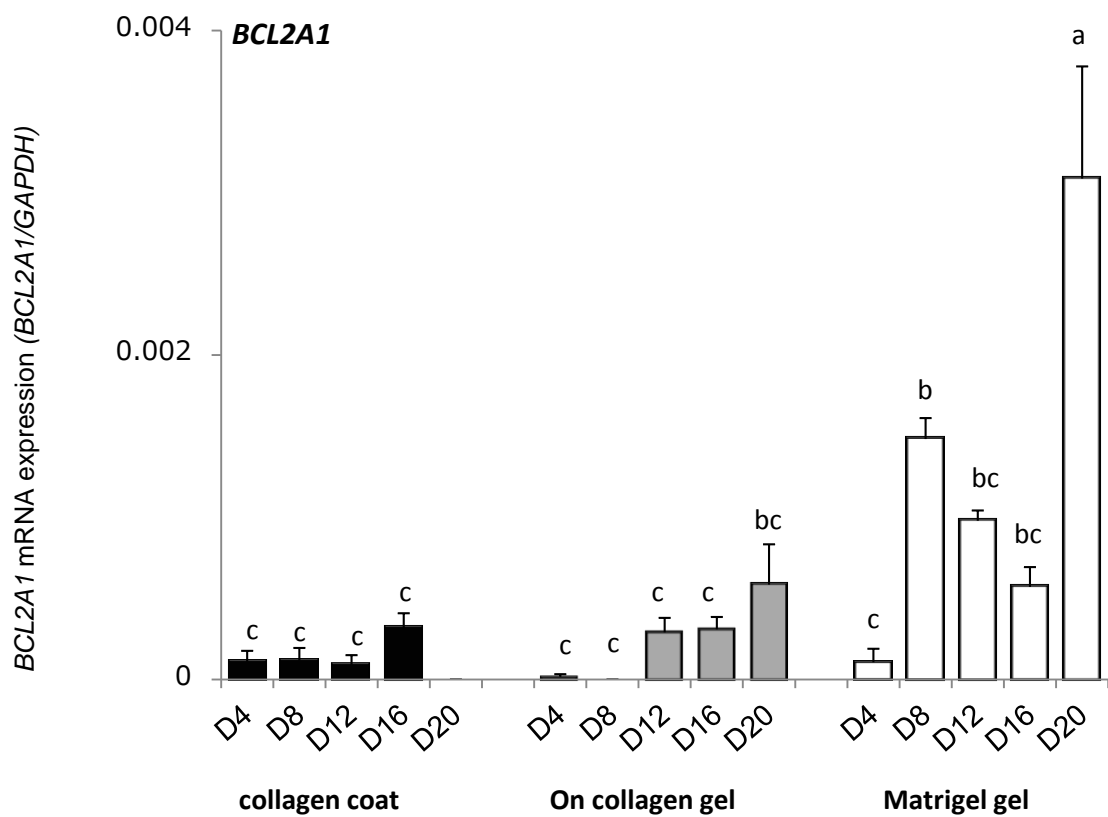

Supplement: Additional file 2: Figure S2 — TNF-α and BCL2A1 expression on different substrates. BT-C was culture on three different substrates, collagen coat (control), collagen gel, and Matrigel, for 20 days. Expression levels of tumor necrosis factor α (TNF-α) and BCL2-related protein A1 (BCL2A1) were normalized to the expression of GAPDH, measured in the corresponding RNA preparation. The black bars, gray bars, and white bars represent the control, collagen gel, and Matrigel cultures, respectively. The different letters indicate a significant difference compared with the control (P < 0.05). [file 1477-7827-12-55-S2.pdf]
